# Searching for nothing: placing zero on the temporal continuum

**DOI:** 10.1007/s10071-023-01770-5

**Published:** 2023-03-27

**Authors:** Carlos Pinto, Sara Salgado

**Affiliations:** School of Psychology, University of Minho, Campus de Gualtar, 4710 Braga, Portugal

**Keywords:** Timing, Generalization, Discrimination learning, Key peck, Pigeons

## Abstract

Generalization allows responses acquired in one situation to be transferred to similar situations. For temporal stimuli, a discontinuity has been found between zero and non-zero durations: responses in trials with no (or 0-s) stimuli and in trials with very short stimuli differ more than what would be expected by generalization. This discontinuity may happen because 0-s durations do not belong to the same continuum as non-zero durations. Alternatively, the discontinuity may be due to generalization decrement effects: a 0-s stimulus differs from a short stimulus not only in duration, but also in its presence, thus leading to greater differences in performance. Aiming to reduce differences between trials with and without a stimulus, we used two procedures to test whether a potential reduction in generalization decrement would bring performance following zero and non-zero durations closer. In both procedures, there was a reduction in the discontinuity between 0-s and short durations, supporting the hypothesis that 0-s durations are integrated in the temporal subjective continuum.

## Introduction

When a response to a stimulus is learned, other stimuli, similar to the one trained, will also tend to elicit responding. The greater the similarity, the stronger this effect—generalization—will be. For instance, pigeons that learned to respond in the presence of a 1000-Hz tone to receive food (and received no food in the absence of that tone), when exposed to other tone frequencies, responded at a high rate when the tones were close to the trained frequency and responded less the greater the difference to 1000 Hz was (Jenkins and Harrison 1960). This pattern of responding is known as a generalization gradient, and the decrease in generalization with greater disparity between trained and non-trained stimuli is called generalization decrement. Generalization is a widespread phenomenon, observed across several dimensions, such as with visual stimuli (e.g., Blough 1967; Guttman and Kalish 1956; Wright 1972), or in numerical (e.g., Fetterman 1993; Pinto and Mota 2022; Rilling 1967) or temporal (e.g., Church and Gibbon 1982; Spetch and Cheng 1998; Vieira de Castro and Machado 2012) discriminations. Generalization is considered to play an important role in categorization learning of natural and associative concepts (e.g., Herrnstein et al. 1976; Katz et al. 2007; Wasserman et al. 1992), and has also been discussed as influencing abstract-concept learning (e.g., Katz and Wright 2021; Premack 1978; Wright and Katz 2007).

Of interest to the present paper is that, along the temporal continuum, there is a case that appears to stray from what is expected by generalization: 0-s stimulus (in other words, trials where no stimulus is presented). It has been assumed that a 0-s duration belongs to the same continuum as non-zero durations (e.g., Kraemer et al. 1985; Sherburne et al. 1998); to illustrate, consider a matching-to-sample task where an animal must discriminate between a short and a long stimulus (known as samples) by choosing between two options (known as comparisons). One comparison is correct following the short sample, and the other comparison is correct following the long sample. When faced with a 0-s, or no-sample trial, the animal should select the “short” comparison because of generalization: a 0-s stimulus would be closer to the shorter of the two learned durations.

**Fig. 1:**
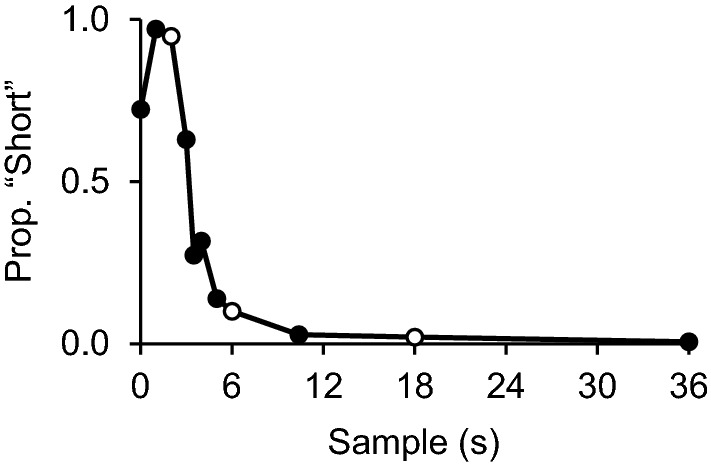
Mean proportion of choices of the comparison associated with short samples as a function of sample duration in a generalization test (*n* = 6). The white data points identify the trained durations (2 s, 6 s and 18 s). [Data from Pinto and Machado (2015)]

Additionally, generalization gradients following this type of temporal discriminations approach the shape of a step function, in that choices following a stimulus outside the trained range tend to be similar to choices following the trained sample closest to that stimulus. That is, for stimuli shorter than the short sample there is a strong preference for the “short” comparison and for stimuli longer than the long sample there is a strong preference for “long”, with little to no generalization decrement (e.g., Spetch and Cheng 1998; Vieira de Castro and Machado 2012). This can be seen in Fig. 1: Pinto and Machado (2015) trained pigeons to choose one comparison following 2-s samples and other comparison following 6 and 18-s samples. In a subsequent generalization test, choices following 18- and 36-s samples were similar; even though 36 s is reasonably distant from 18 s, there was no generalization decrement. Similarly, choices following 0-s samples should be similar to 2-s samples.

A preference for “short” has been consistently found in no-sample tests; however, not as strong as predicted by generalization. A no-sample trial would be expected to generate a strong preference for “short” (0 s is the shortest a sample can be), but typically the proportion of “short” choices ranges from 0.60 to 0.80 (Church 1980; Pinto et al. 2017; Pinto and Machado 2015; 2017; Pinto and Sousa 2021; Spetch and Wilkie 1983). Figure 1 illustrates this typical finding: choices were similar following 2 s (leftmost white data point) and 1 s, a shorter duration. However, there was a discontinuity between the shorter samples and a 0-s sample: preference for “short” decreased markedly in no-sample trials (see also Pinto and Mota 2022; Pinto and Sousa 2021).

The discontinuity in the generalization gradient may suggest that a 0-s stimulus is not part of the same continuum as non-zero durations or, alternatively, may be the combined result of stimulus generalization (that would lead to a strong preference for “short”) and generalization decrement (that would lead to choices toward chance levels). The generalization decrement would be significant because the disparity between training and no-sample trials is, by necessity, pronounced: no-sample trials do not differ only in the *duration* of a stimulus, but in its *presence*. To clarify this issue, the goal of the present study was to make training trials and no-sample test trials more similar, so that a putative effect of generalization decrement would be reduced.

On the one hand, if there is a qualitative difference between zero and non-zero durations (and they do not belong to the same continuum), making training and testing more similar should not yield results different to what has been typically found. On the other hand, if zero and non-zero durations do belong to the same continuum, and the discontinuity between them in generalization tests is due to generalization decrement, making training and testing more similar should reduce that discontinuity.

To reduce the difference between training and testing, we employed empty intervals; that is, no stimulus was presented during the to-be-timed duration, which was bounded instead by “start” and “stop” time markers. If the pigeons learn to time intervals during which no stimulus is present, the absence of a stimulus in a no-sample test could lead to less generalization decrement.

**Fig. 2:**
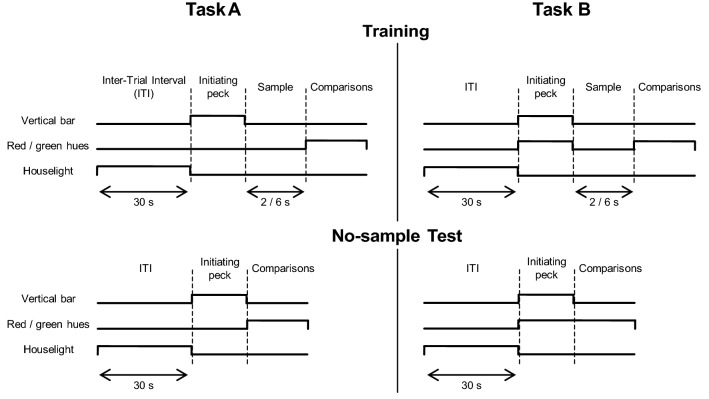
Schematic of the stimuli presented in a trial in training (top panels) and no-sample test (bottom panels) in Task A (left column) and B (right column). Each line was raised whenever its corresponding stimulus was turned on. The duration of presentation of the vertical bar and red/green hues was controlled by the animals

We used two tasks, depicted in Fig. 2. In Task A (Fig. 2, left panels), following a 30-s, houselight-illuminated, inter-trial interval (ITI), a white vertical bar on a black background was presented on the central key. When this key was pecked, it turned off, marking the beginning of the sample interval. When the sample duration elapsed, the two lateral keys were illuminated, one with a red hue, the other with a green hue, and the animals had then to choose one of the keys. Therefore, the sample duration was the interval between the offset of the vertical bar and the onset of the colored hues. In the no-sample test, the comparisons were presented immediately following the removal of the vertical bar (Fig. 2, bottom left panel). Even though the presentation of the comparisons was immediate, given the transition between stimuli, these trials could be perceived as having a very short sample instead of no sample at all. Therefore, a second group of pigeons learned a slightly different task, where there would be no stimuli turning on to signal the end of the sample.

In Task B (Fig. 2, right panels), following the ITI, all three keys were illuminated, the lateral keys with a red and a green hue, and the central key with a vertical bar. These stimuli were presented until the central key was pecked once. In a training trial (Fig. 2, top right panel), all keys were turned off, and the sample interval began. The lateral keys were illuminated once more to signal the end of the sample, and allowing the pigeons to choose a comparison. As in Task A, the to-be-timed interval was spent in darkness, but in this case the “start” time marker was the offset of all three keys. In a no-sample trial (Fig. 2, bottom right panel), after the peck that initiated a trial only the center key turned off: the comparisons were immediately available for choice. Even though having the comparisons available since the beginning of the trial could reduce the chances of a trial with no sample being interpreted as one with a very short sample, the no-sample test introduced a change in trial events that could generate more generalization decrement than Task A: the pigeons were trained to see the red and green keys turn off and then turn on again, and in testing they were never turned off.

A no-sample test, by necessity, introduces changes that may result in considerable generalization decrement, making an ideal test difficult to find. The two tasks here presented are attempts to reduce this issue and, taken together, may allow a better assessment of whether the absence of a temporal stimulus is integrated in the same continuum as non-zero durations.

## Methods

### Subjects

12 pigeons (*Columba livia*) participated in this study, divided into two groups of six. In previous studies (listed in Table 1), when comparing no-sample and short-sample trials, the average effect size (*d*_*z*_) was 1.93 (range = 1.20–2.62). For that effect size, an a priori power analysis (calculated with G*Power), for a power of 0.8, recommends a sample size of 5. The birds were maintained at 85% of their free-feeding body weight and had free access to water and grit in their individual home cages. The colony room had a 13:11-h light/dark cycle (lights on at 08:00), and its temperature was kept between 20 and 22 °C. The experiment was conducted once a day, at approximately the same time for each pigeon, 6 days a week. All pigeons had experience with temporal discriminations in matching-to-sample tasks.

**Table 1:** Choices of the “short” comparison in short-sample and no-sample trials

Study	Proportion “short” (range)			
	Short sample	No sample	Difference	Equivalent?
Pinto et al. (2017)	0.94 (0.88–0.98)	0.73 (0.45–0.93)	0.20**	No (*p* = 0.737)
Pinto and Machado (2015)	0.95 (0.92–0.99)	0.72 (0.55–0.80)	0.23**	No (*p* = 0.942)
	0.97 (0.94–0.99)	0.68 (0.50–0.77)	0.29**	No (*p* = 0.981)
Pinto and Machado (2017)	0.94 (0.91–0.97)	0.68 (0.47–0.83)	0.26***	No (*p* = 0.989)
Pinto and Machado (2023)	0.92 (0.82–0.99)	0.62 (0.21–0.89)	0.30***	No (*p* = 0.985)
Task A	0.85 (0.77–0.96)	0.91 (0.70–1.00)	− 0.05	Yes (*p* = 0.023)
Task B	0.87 (0.80–0.94)	0.81 (0.65–0.95)	0.06	Yes (*p* = 0.028)

In all studies the short sample lasted 2 s

**p < 0.01; ***p < 0.001

### Apparatus

Four operant chambers were used: one homemade chamber and three LVE (Lehigh Valley Electronics) chambers. The homemade chamber measured 31 × 33 × 33 cm (height × length × width). On the intelligence panel, three response keys, 2.5 cm in diameter, were distributed horizontally, 9 cm apart (center to center). The bottom edge of the keys was 21 cm above the wire mesh floor. Stimuli were presented in each key through a 12-stimulus IEE (Industrial Electronics Engineers) in-line projector—a 28-V, 0.1-A lamp illuminated each stimulus. Reinforcement, mixed grain, was delivered through a LVE food hooper, with a 6 × 4.5 cm (w × h) opening, centered horizontally on the response panel, 6.5 cm above the floor. When activated, A 28-V, 0.04-A light illuminated the hopper opening. On the wall opposite the intelligence panel, 27.5 cm above the floor, there was a 28-V, 0.1-A houselight. The operant chamber was enclosed in a PVC sound-attenuating cubicle (Med Associates, ENV-018 V) equipped with an exhaust fan.

The LVE chambers measured 34 × 35 × 31 cm (h × l × w), and were equipped with three circular response keys, arranged horizontally, 9 cm apart, center to center. The keys were 2.5 cm in diameter and 22.5 cm above the wire mesh floor. Each key was equipped with a 12-stimulus IEE in-line projector (each stimulus was illuminated with a 28-V, 0.1-A lamp). The opening of the LVE food hopper, centered horizontally on the intelligence panel, 8.5 cm above the floor, was 6-cm wide × 5-cm high. When the hopper was raised to allow access to food, a 28-V, 0.04-A light illuminated the opening. A 28-V, 0.1-A houselight, on the wall opposite the intelligence panel, provided general illumination. An exhaust fan circulated air through the chamber and masked outside noises. A computer running the ABET II software (Lafayette Instrument Company) controlled the experiment and recorded the data.

### Procedure

At the start of every session pigeons were at 85% of their free-feeding weight, and reinforcement duration was adjusted individually to minimize feeding outside the sessions. Half the birds ran Task A, half Task B.

### Task A

#### Training

The pigeons learned a symbolic matching-to-sample task with samples that differed in duration. A session began with a 30-s, houselight-illuminated, inter-trial interval (ITI). Following the ITI, the houselight was turned off and a white vertical bar on a black background was presented in the center key. This stimulus was presented until the center key was pecked once: the key was then turned off and the sample interval (spent in darkness) began. After the sample duration (2 or 6 s) elapsed, the two side keys were illuminated, one with a green hue and the other with a red hue (comparisons). One comparison was correct following the 2-s sample, and the other following the 6-s sample (the correct comparison following each sample was counterbalanced across subjects). After a peck to either comparison, both keys were turned off. If the choice was correct, reinforcement was delivered and, after that, a new ITI started. If the choice was incorrect, the ITI started immediately. To facilitate acquisition, we employed a correction procedure where a trial was repeated following an incorrect response; after three consecutive incorrect responses, only the correct comparison was presented. Reinforcement durations varied between 2 and 4 s. The top left panel of Fig. 2 presents a diagram of the events in a training trial.

Excluding correction trials, a session comprised 64 trials, which were divided into two 32-trial blocks. Each block contained 16 2-s sample trials and 16 6-s sample trials, and were presented in random order. The blocks were used to ensure a more balanced distribution of trials throughout the session. The location of the comparisons was counterbalanced within a block so that each was presented the same number of times in each side key. Training could last between 15 and 30 sessions, and was completed when the pigeon met a criterion of at least 80% correct responses to each sample in a session (excluding correction trials), for three consecutive sessions.

#### Generalization test

Each session included 76 trials: 56 training (28 2-s sample + 28 6 s sample) and 20 generalization trials. A generalization trial was identical to a training trial, with the exception that either no sample was presented or it lasted 0.67, 1.15, 3.46, or 10.39 s. In a no-sample trial, the comparisons were presented immediately following the trial-initiating peck (bottom left panel of Fig. 2). The 3.46-s sample is at the geometric mean of 2 and 6, the two trained samples, so chosen to be equally discriminable from both, as per Weber’s law. The remaining samples were selected to maintain the same ratio between all sample durations (1:1.73). Half of the generalization trials were reinforced, randomly: overall, a pigeon could receive reinforcement on, at most, 66 of the 76 (~ 87%) trials. The trials were presented in two 38-trial blocks, each containing 28 training trials (14 of each trained sample) and 10 generalization trials (two per sample), randomly distributed. Testing lasted for five sessions.

### Task B

#### Training

The procedure was similar to Task A, with the exception that, at trial onset, in addition to a vertical bar on the center key, a red and a green hue were presented in each of the side keys. Pecks on the side keys were ignored at this stage. A peck on the center key turned off all three stimuli, marking the beginning of the sample interval. As in Task A, the end of the sample was signaled by the illumination of the side keys with a red and a green hue (the top right panel of Fig. 2 illustrates the stimulus presentation). The session structure was the same as in Task A. Reinforcement durations varied between 2.5 and 3 s.

#### Generalization test

Sessions followed the same structure as in Task A. In a no-sample trial, following the ITI, all three keys were illuminated, the center key with a vertical bar and the side keys with a red and a green hue. A peck on the center key turned off only its light, with the side keys remaining on until a choice was made (bottom right panel of Fig. 2). The remaining generalization trials followed the same structure as regular training trials.

### Data analysis

We analyzed choice behavior following the different sample durations. Pigeons responded by pecking on plastic keys, and the pecks were recorded by the ABET II software (Lafayette Instrument Company). To compare choices following 3.46-s samples with chance levels, 95% Confidence Intervals were calculated. Generalization data was modeled with a mixed logistic regression model (Gallucci 2019), with post-hoc comparisons (with Bonferroni correction) to contrast choices following 0-s samples with 0.67 and 2-s samples, using the jamovi software for Windows (version 2.3.21). Equivalence tests were run via the “two one-sided tests” (TOST) procedure (e.g., Lakens 2017). To set the upper and lower equivalence bounds (− Δ_L_ and Δ_U_), the smallest effect sizes of interest were calculated via a sensitivity analysis in G*Power (version 3.1.9.7; Faul et al. 2007), with power set to 80% and alpha to 0.05 (Lakens 2014). Choice difference between no-sample and short-sample trials in the present study was compared with a previous study via a two-tailed independent samples *t* test (with Type-1 error rate set at 0.05), with the standardized mean difference effect size for independent observations (*d*_*S*_, e.g., Cohen 1988, p. 48; Lakens 2013) as measure of effect size. A Welch’s *t* test was used when sample sizes were not equal.

## Results

### Training

Five of the six pigeons in Task A met the training criterion, taking an average of 23 sessions (range: 15–30) to complete training. For these birds, average matching accuracy in the last three sessions of training was 0.88 (range: 0.83–0.98) for 2-s samples and 0.89 (range: 0.84–0.98) for 6-s samples. One pigeon, despite small fluctuations that precluded accuracy from stabilizing above 80% for three consecutive sessions, was close to criterion—by the last three sessions of training, average accuracy was 0.79 for both samples—and advanced to testing. Four of the six pigeons in Task B met the training criterion in an average of 20 sessions (range: 15–24). By the last three sessions of training, average matching accuracy for these pigeons was 0.90 (range: 0.88–0.93) for 2-s samples and 0.92 (range: 0.88–0.97) for 6-s samples. Of the other two pigeons, one, similarly to Task A, showed small fluctuations around the criterion, but learned the discrimination (average accuracy was 0.81 for both samples in the last three sessions of training) and went into testing. The last pigeon failed to consistently reach 0.80 accuracy to the long samples (on the 30th session accuracy was below 0.70) and did not advance into testing.

**Fig. 3:**
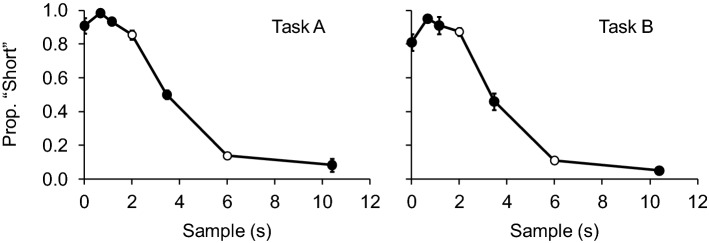
Mean (with SEM) proportion of choices of the comparison associated with short samples as a function of sample duration in the generalization test of Task A (left panel, *n* = 6) and Task B (right panel, *n* = 5). The white data points identify the trained durations

Figure 3 shows the results of the generalization tests. In both tasks, a typical generalization gradient was observed: there was a strong preference for the “short” comparison following the shorter samples, and for the “long” comparison following the longest sample. For the sample at the geometric mean of the trained durations (3.46 s), choices approached chance levels: for Task A it was exactly 0.50, and for Task B it was 0.46, a value not significantly different from 0.50: 95% Confidence Interval = [0.36–0.56]). An equivalence test indicated that the observed effect size (0.34) was significantly within the bounds of *d* = − 1.68 and *d* = 1.68, (*t*(4) = 3.01, *p* = 0.02).

For 0-s samples, both generalization gradients show a small decrease in choices of “short” when compared with the shorter samples. First, when compared with the shortest non-zero sample (0.67 s), choices following 0-s samples were not statistically different in Task A (*z* = 2.29, *p* = 0.461) or task B (*z* = 2.86, *p* = 0.089). Regarding equivalence, while for Task A choices following 0-s and 0.67-s samples were statistically equivalent (the observed effect size, *d*_*z*_ = 0.6, was within the equivalence bounds of *d*_*z*_ = − 1.43 and *d*_*z*_ = 1.43, *t*(5) =  − 2.05, *p* = 0.048), that was not the case for Task B (*d*_*z*_ = 1.17 was not significantly within the equivalence bounds of *d*_*z*_ = − 1.68 and *d*_*z*_ = 1.68, *t*(4) = − 1.14, *p* = 0.159). Second, when compared with the trained 2-s sample, choices following 0-s samples were not statistically different in Task A (*z* = − 1.58, *p* = 1) or task B (*z* = 1.71, *p* = 1). Choices following 0-s and 2-s samples were equivalent both in Task A (*d*_*z*_ = − 0.35; Δ_L_ = − 1.43, Δ_U_ = 1.43; *t*(5) = 2.65, *p* = 0.023) and Task B (*d*_*z*_ = 0.49; Δ_L_ = − 1.68, Δ_U_ = 1.68; *t*(4) = − 2.67, *p* = 0.028).

## Discussion

Two generalization tests were run to clarify whether the absence (duration = 0 s) or presence (duration > 0 s) of an event are considered as qualitatively different or only as differing quantitatively along the same temporal continuum. In previous experiments, these two possibilities may have been difficult to distinguish due to generalization decrement effects, so the tasks in the present work aimed to diminish those effects, by reducing the dissimilarities between trials with and without samples.

Traditionally, preference for the “short” comparison in no-sample trials is not as pronounced as in short-sample trials, resulting in a discontinuity in the generalization gradient (this discontinuity can be seen in the inverted U shape on the left tail of the generalization gradient shown in Fig. 1). In the present experiment, that inverted U shape could still be seen (more so in Task B), but it was not as pronounced as in previous experiments. Even though, in both tasks, choices following no sample did not differ statistically from choices following short samples, in Task B choices following 0-s and 0.67-s samples were not statistically equivalent, which makes this comparison less conclusive. As mentioned in the Introduction, Task B may induce greater generalization decrement, and the fact that the discrepancy between no sample and short samples was more pronounced in this task is consistent with a generalization account. The finding that choices following no-sample and short-sample trials become more similar as the trials themselves are made more similar is consistent with the possibility that zero and non-zero durations share the same continuum—and that previous differences in performance were due to generalization decrements stemming from how the trials were arranged.

Table 1 compares choices  in short-sample and no-sample trials in previous experiments (to which we had access to the full data set) and in the two tasks of the present study. All the previous experiments listed employed filled intervals (a stimulus was presented throughout the duration being timed). First, baseline matching accuracy (leftmost column) is slightly lower in the present empty-interval tasks than in the filled-interval tasks. This result replicates Mantanus (1981), who found that discriminations of filled durations were more accurate. Second, and of most importance to the present work, the difference in proportion of “short” choices between short and no-sample trials is presented in the “Difference” column (with corresponding equivalence tests in the rightmost column). While these differences ranged from 0.20 to 0.30 in the filled-interval studies (choices were not equivalent and statistically different in all cases), they fell to values close to 0.05 in the present study (choices were statistically equivalent and not different). Among the studies listed in Table 1, the generalization test from Pinto and Machado (2015; second row, also depicted in Fig. 1) is procedurally the most similar to the present experiment. To complement this analysis, we directly compared tasks A and B with the “No-sample Test” condition from that study, and found that the difference between no-sample and short-sample trial was significant smaller in both Task A (*t*(10) = 3.77, *p* = 0.004, *d*_*S*_ = 2.18) and Task B (*t*(7.7) = 2.35, *p* = 0.048, *d*_*S*_ = 1.44). By making training trials more similar to no-sample test trials, differences in performance were reduced.

However, simply employing empty intervals is not sufficient to reduce generalization decrement. Empty intervals are usually bounded by specific “start” and “stop” time marker stimuli, which are then removed in no-sample tests: this difference between training and testing conditions may reduce generalization. Santi et al. (1999), using tones as time markers, trained pigeons to discriminate between 3- and 9-s empty intervals. On subsequent no-sample tests, no time markers were presented, but the ITI was lengthened 3 or 9 s. Choice proportions of the “short” comparison were between 0.60 and 0.70, values similar to those found in filled-interval tasks (see Table 1). Grant (2001, Experiment 1) employed red and green keylights as time markers, and sample and comparisons were separated by a delay during training. On no-sample tests, choices approached indifference. Finally, Santi et al. (2003, Experiment 1), signaling the samples with a white square, trained pigeons to discriminate between 2 and 8 s, that could be presented as empty or filled intervals. The two discriminations were learned concurrently (each discrimination with its own set of comparisons), and then, on no-sample tests, no time markers were presented, but the ITI was lengthened 2 or 8 s. When faced with the comparisons associated with empty-interval samples, pigeons predominantly chose the “long” comparison (the proportion of "short" choices was only 0.31). Given the procedural differences between the three studies, it is difficult to integrate their results (as well as compare them with our data), but this variety of outcomes highlights how much task differences may affect performance. By the same token, an experiment that employs the same procedural details as in the present tasks, but featuring filled intervals, may prove informative in ascertaining how 0-s durations fit in the temporal continuum.

Clarifying how much of a result is due to procedural details improves not only our understanding of the process under study—in this case how durations are perceived—, but also allows better-informed decisions on task selection and design. Our findings suggest that previous discrepancies between zero and non-zero samples in generalization tests were not due to a seeming discontinuity between them, but to generalization decrement effects. Results from numerical discriminations, suggesting that honey bees (Howard et al. 2018), crows (Kirschhock et al. 2021), and rhesus monkeys (Merritt et al. 2009) place empty sets (a numerosity of “zero”) in the same continuum as other numerosities help place the present results in a bigger picture: absence vs. presence discriminations can be integrated as part of the same continuum, and this ability is seemingly shared by several species.

## Data Availability

The datasets generated and analyzed during the current study are available in the DataRepositoriUM repository, https://doi.org/10.34622/datarepositorium/JEJHGT

## References

[CR1] Blough DS (1967). Stimulus generalization as signal detection in pigeons. Science.

[CR2] Church R (1980). Short-term memory for time intervals. Learn Motiv.

[CR3] Church RM, Gibbon J (1982). Temporal generalization. J Exp Psychol Anim Behav Process.

[CR4] Cohen J (1988). Statistical power analysis for the behavioral sciences.

[CR5] Faul F, Erdfelder E, Lang A-G, Buchner A (2007). G*Power 3: a flexible statistical power analysis program for the social, behavioral, and biomedical sciences. Behav Res Methods.

[CR6] Fetterman JG (1993). Numerosity discrimination: both time and number matter. J Exp Psychol Anim Behav Process.

[CR7] Gallucci M (2019) GAMLj: General analyses for linear models. [jamovi module]. https://gamlj.github.io/

[CR35] Grant D (2001). Memory for empty time intervals in pigeons. Anim Learn Behav.

[CR8] Guttman N, Kalish HI (1956). Discriminability and stimulus generalization. J Exp Psychol.

[CR9] Herrnstein RJ, Loveland DH, Cable C (1976). Natural concepts in pigeons. J Exp Psychol Anim Behav Process.

[CR10] Howard SR, Avarguès-Weber A, Garcia JE, Greentree AD, Dyer AG (2018). Numerical ordering of zero in honey bees. Science.

[CR11] Jenkins HM, Harrison RH (1960). Effect of discrimination training on auditory generalization. J Exp Psychol.

[CR12] Katz JS, Wright AA (2021). Issues in the comparative cognition of same/different abstract-concept learning. Curr Opin Behav Sci.

[CR13] Katz JS, Wright AA, Bodily KD (2007). Issues in the comparative cognition of abstract-concept learning. Comp Cogn Behav Rev.

[CR14] Kirschhock ME, Ditz HM, Nieder A (2021). Behavioral and neuronal representation of numerosity zero in the crow. J Neurosci.

[CR15] Kraemer PJ, Mazmanian DS, Roberts WA (1985). The choose-short effect in pigeon memory for stimulus duration: subjective shortening versus coding models. Anim Learn Behav.

[CR16] Lakens D (2013). Calculating and reporting effect sizes to facilitate cumulative science: a practical primer for t-tests and ANOVAs. Front Psychol.

[CR17] Lakens D (2014). Performing high-powered studies efficiently with sequential analyses: sequential analyses. Eur J Soc Psychol.

[CR18] Lakens D (2017). Equivalence tests: a practical primer for t tests, correlations, and meta-analyses. Soc Psychol Personal Sci.

[CR36] Mantanus H (1981). Empty and filled interval discrimination by pigeons. Behav Anal Lett.

[CR19] Merritt DJ, Rugani R, Brannon EM (2009). Empty sets as part of the numerical continuum: conceptual precursors to the zero concept in rhesus monkeys. J Exp Psychol Gen.

[CR20] Pinto C, Machado A (2015). Coding in pigeons: multiple-coding versus single-code/default strategies. J Exp Anal Behav.

[CR21] Pinto C, Machado A (2017). Unraveling sources of stimulus control in a temporal discrimination task. Learn Behav.

[CR22] Pinto C, Machado A (2023) Trade-offs in joint stimulus control in a temporal discrimination task (**Manuscript submitted for publication**) 10.1007/s10071-017-1130-6 28914387

[CR23] Pinto C, Mota M (2022). Number-of-responses matching in pigeons (*Columba livia*): choice biases following delay and no-sample tests. Behav Process.

[CR24] Pinto C, Sousa A (2021). Choice biases in no-sample and delay testing in pigeons (*Columba livia*). Anim Cogn.

[CR25] Pinto C, Fortes I, Machado A (2017). Joint stimulus control in a temporal discrimination task. Anim Cogn.

[CR26] Premack D, Hulse SH, Fowler H, Honig WK (1978). On the abstractness of human concepts: why it would be difficult to talk to a pigeon. Cognitive processes in animal behavior.

[CR27] Rilling M (1967). Number of responses as a stimulus in fixed interval and fixed ratio schedules. J Comp Physiol Psychol.

[CR340] Santi A, Hornyak S, Miki A (2003). Pigeons’ memory for empty and filled time intervals signaled by light. Learn Motiv.

[CR37] Santi A, Ross L, Coppa R, Coyle J (1999). Pigeons’ memory for empty time intervals marked by visual or auditory stimuli. Anim Learn Behav.

[CR28] Sherburne LM, Zentall TR, Kaiser DH (1998). Timing in pigeons: the choose-short effect may result from pigeons’ “confusion” between delay and intertrial intervals. Psychon Bull Rev.

[CR29] Spetch ML, Cheng K (1998). A step function in pigeons’ temporal generalization in the peak shift task. Anim Learn Behav.

[CR30] Spetch ML, Wilkie DM (1983). Subjective shortening: a model of pigeons’ memory for event duration. J Exp Psychol Anim Behav Process.

[CR31] Vieira de Castro AC, Machado A (2012). The interaction of temporal generalization gradients predicts the context effect. J Exp Anal Behav.

[CR32] Wasserman EA, DeVolder CL, Coppage DJ (1992). Non-similarity-based conceptualization in pigeons via secondary or mediated generalization. Psychol Sci.

[CR33] Wright AA (1972). Psychometric and psychophysical hue discrimination functions for the pigeon. Vis Res.

[CR34] Wright AA, Katz JS (2007). Generalization hypothesis of abstract-concept learning: learning strategies and related issues in *Macaca mulatta*, *Cebus apella*, and *Columba livia*. J Comp Psychol.

